# Harnessing cellular immunity for next-generation vaccines against respiratory viruses: mechanisms, platforms, and optimization strategies

**DOI:** 10.3389/fimmu.2025.1618406

**Published:** 2025-08-13

**Authors:** Keda Chen, Jutao Hu, Jiaxuan Li, Guangshang Wu, Xiaotian Tie, Hao Wu, Hongyu Li, Jianhua Li, Yanjun Zhang

**Affiliations:** ^1^ Key Laboratory of Artificial Organs and Computational Medicine of Zhejiang Province, Shulan International Medical College, Zhejiang Shuren University, Hangzhou, China; ^2^ School of Basic Medical Sciences, Zhejiang Chinese Medical University, Hangzhou, China; ^3^ Zhejiang Key Laboratory of Public Health Detection and Pathogenesis Research, Department of Microbiology, Zhejiang Provincial Center for Disease Control and Prevention, Hangzhou, China

**Keywords:** upper respiratory tract infection, vaccine, humoral immunity, cellular immunity, vaccine optimization

## Abstract

Respiratory tract infections, such as influenza, respiratory syncytial virus (RSV) infection, and COVID-19, remain a persistent threat to global public health due to their high transmissibility and disease burden. Vaccination, as a key preventive strategy, not only reduces the risk of infection but also blocks transmission by activating adaptive immunity. While traditional vaccine evaluations have primarily focused on humoral immunity, growing evidence highlights the critical role of T lymphocyte-mediated cellular immunity in clearing virus-infected cells, establishing long-term immune memory, and responding to viral mutations. This review systematically summarizes the cellular immune responses induced by vaccines against respiratory tract infections and their correlation with protective efficacy. It also outlines evaluation methodologies such as flow cytometry, providing a theoretical foundation for optimizing vaccine design and assessment, and advancing the development of effective, broad-spectrum vaccines.

## 1 Introduction

Upper respiratory tract viruses-such as influenza virus, SARS-CoV-2, and respiratory syncytial virus (RSV)—pose a continued threat to global public health due to their high transmissibility and potential for mutation. According to estimates by the World Health Organization, respiratory infections account for approximately 1 billion cases annually, including 3 to 5 million severe cases and 290,000 to 650,000 related deaths (1). Among them, influenza virus, adenovirus, and the novel coronavirus are the most prominent representatives (2). The 1918 Spanish flu was characterized by both high mortality and high transmissibility, resulting in the deaths of over 50 million people worldwide (3). The COVID-19 outbreak that began in 2019 rapidly evolved into a global public health crisis. As of July 7, 2024, a total of 775,754,322 confirmed COVID-19 cases have been reported to the WHO (World Health Organization), including 7,053,902 deaths worldwide. Furthermore, WHO reports that by 2025, respiratory syncytial virus (RSV) is expected to cause approximately 33 million cases of acute lower respiratory tract infections annually, resulting in over 3 million hospitalizations and 59,600 in-hospital deaths among children under the age of five.

**Figure 1:**
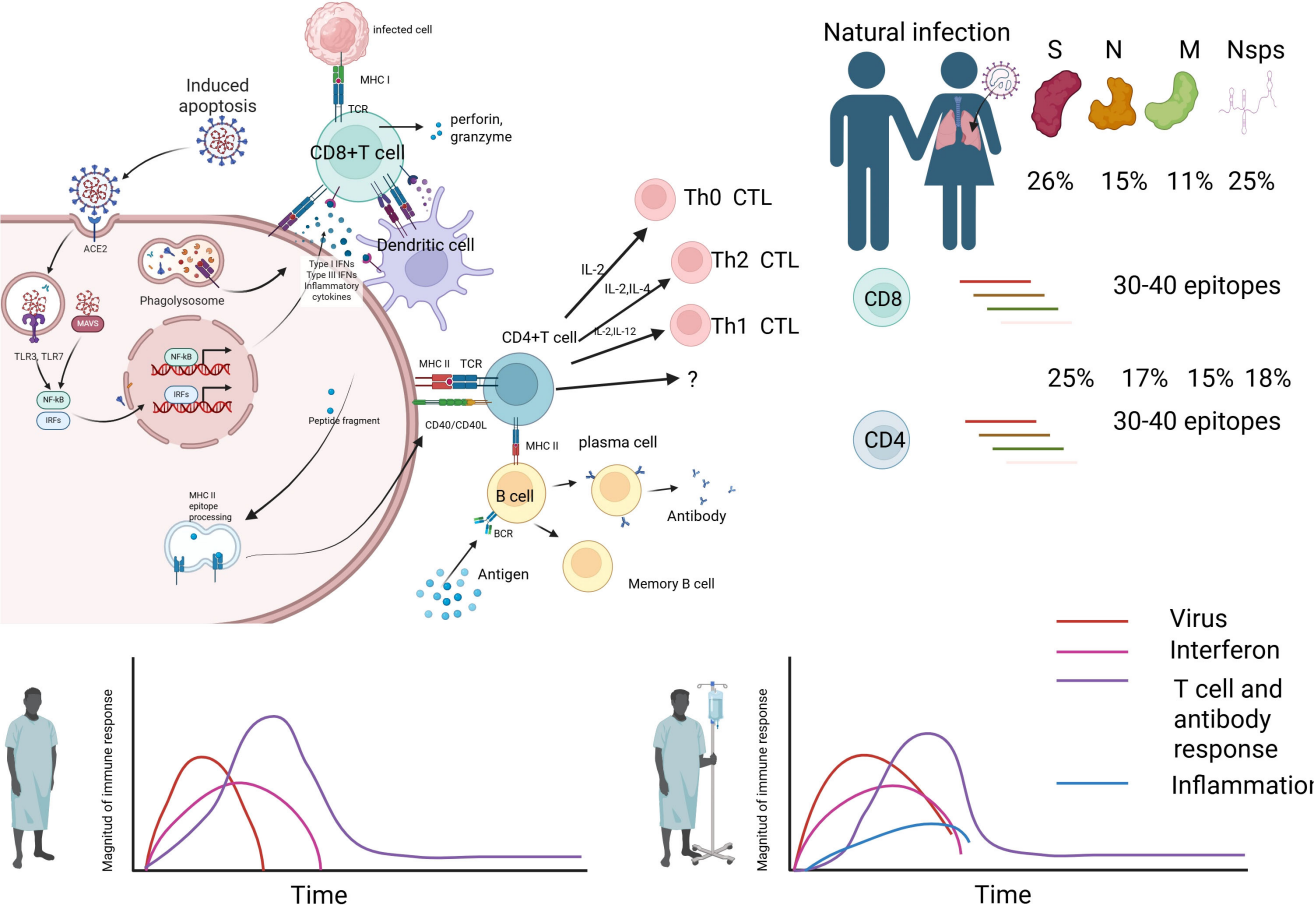
Immune response mechanism during natural infection. Image 1 illustrates the immune response mechanism during natural infection. The virus infects cells via the ACE2 receptor, leading to phagocytosis by macrophages (MФ), which release inflammatory mediators such as NF-κB, IRFs, and IFNs, inducing Type I IFNs and inflammatory cytokines. Infected cells present antigens via MHC class I molecules, activating CD8+ T cells (CTLs) to release perforin and granzyme, targeting infected cells for destruction. Dendritic cells (DCs) present antigens via MHC class II molecules, activating CD4+ T cells (Th0 CTL), which differentiate into Th1 and Th2 cells, regulating immunity through IL-2, IL-12, IFN-γ, and IL-2, IL-4, respectively. B cells, with help from CD4+ T cells, differentiate into plasma cells to produce antibodies and form memory B cells for future immunity. The bar chart on the right indicates the proportion of T cell and antibody responses to 30–40 epitopes on viral proteins S, N, M, and Nsps, with 26%, 15%, 11%, and 25% for S protein, and 25%, 17%, 15%, and 18% for others, respectively. The lower line graph depicts the dynamics of virus (red), interferon (pink), T cell and antibody responses (purple), and inflammation (blue) over time, reflecting the intensity and duration of the immune response.

Currently, vaccination against corresponding upper respiratory tract viruses is one of the key measures for preventing infection, reducing severe cases, and minimizing related deaths (4). Over the years, vaccines targeting various pathogens have saved hundreds of millions of lives. There is an increasing recognition of the immense potential of vaccines in controlling disease outbreaks and preventing severe cases. However, traditional vaccine evaluation systems have long focused on humoral immunity (such as neutralizing antibody titers), neglecting the crucial role of cellular immunity. Recent studies have revealed that T lymphocyte-mediated cellular immunity not only directly eliminates virus-infected host cells but also provides long-lasting protection by forming tissue-resident memory T cells (TRM) and circulating memory T cells (TCM). Furthermore, it demonstrates unique advantages in responding to viral antigenic drift or escape mutations (5, 6). For example, studies on SARS-CoV-2 variants have shown that, although the potency of neutralizing antibodies may decrease, T cells’ recognition of conserved epitopes (such as the S2 subunit and nucleocapsid protein) can still effectively reduce the risk of severe disease (7–9). This finding highlights the strategic value of cellular immunity in the design of broad-spectrum vaccines (**Figure 1**).

**Figure 2:**
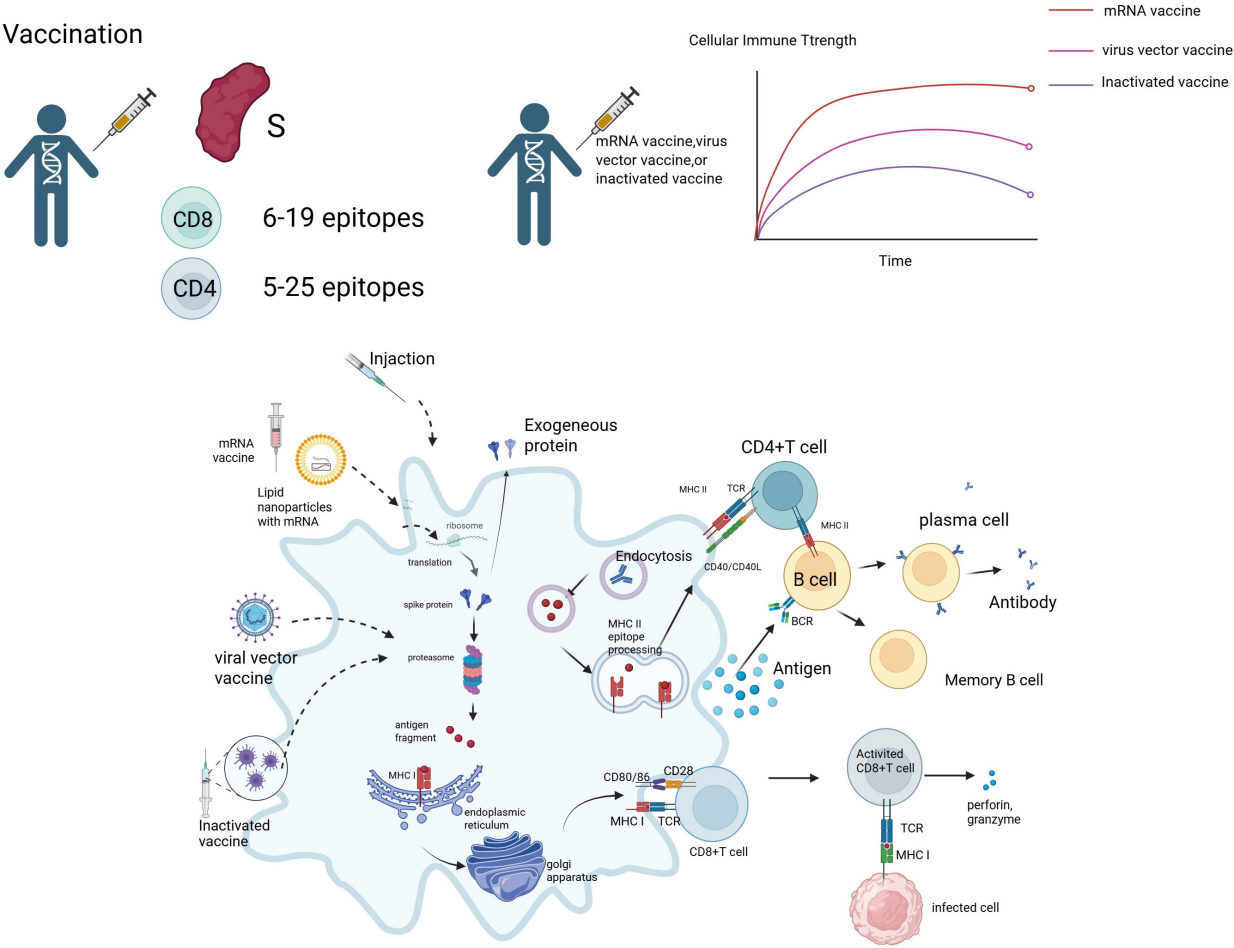
Vaccine-induced immune response mechanism. Image 2 depicts the immune response induced by mRNA vaccines, viral vector vaccines, and inactivated vaccines. mRNA vaccines deliver mRNA via lipid nanoparticles (LNPs), translating into S protein, while viral vector vaccines express S protein through endogenous proteins, and inactivated vaccines provide antigen fragments directly. Antigen-presenting cells (APCs) activate CD4+ T cells via MHC class II molecules, which differentiate into Th1 and Th2 cells, regulating immunity through IL-2, IL-12, and IL-2, IL-4, respectively. CD4+ T cells assist B cells in differentiating into plasma cells, producing antibodies against the S protein and forming memory B cells. MHC class I molecules activate CD8+ T cells (CTLs), which release perforin and granzyme to kill infected cells. Vaccine-induced CD8+ T cells recognize 6–19 epitopes, while CD4+ T cells recognize 5–25 epitopes. The line graph on the right shows the cellular immune strength over time for mRNA vaccines (red), viral vector vaccines (pink), and inactivated vaccines (purple), indicating that mRNA vaccines induce a stronger early response that gradually stabilizes, while other vaccines produce a lower but more sustained response.

However, there are significant differences among various vaccine platforms in inducing cellular immunity. Traditional inactivated vaccines, while highly safe, primarily activate CD4+ Th2 responses and are relatively weak in activating CD8+ cytotoxic T cells (CTLs) (10). In contrast, mRNA vaccines efficiently induce multi-epitope-specific CTLs and TRMs through endogenous antigen presentation. However, their durability and reliance on cold-chain storage remain significant challenges (11, 12). Viral vector vaccines, on the other hand, mimic the natural infection pathway, providing both mucosal and systemic immunity, but may be affected by pre-existing immunity (**Figure 2**).

(13, 14) These differences not only determine the protective efficacy of vaccines but also provide direction for optimization: how to coordinate mucosal local immunity and systemic immunity through adjuvant modulation, antigen targeting, and innovations in delivery systems has become a central issue in the development of next-generation vaccines.

Currently, the frequent emergence of viral variants, the low response rates in elderly individuals and immunocompromised patients, as well as the bottlenecks in mucosal immunity induction technologies, further complicate vaccine design. This paper elucidates the core mechanisms of cellular immunity in the defense against upper respiratory tract viruses, systematically compares the efficacy differences in cellular immunity induction across different vaccine platforms, and proposes optimization strategies based on the synergy between mucosal and systemic immunity, along with future directions to address clinical challenges. By integrating cutting-edge research, the goal is to provide a theoretical framework and technical insights for developing efficient, broad-spectrum vaccines against upper respiratory tract viruses, driving the paradigm shift from “population protection” to “precision immunization.”

## 2 Interaction between upper respiratory tract viruses and host cell immunity

### 2.1 Viral escape mechanisms and the defensive role of cellular

#### 2.1.1 Immunity influenza virus

For influenza virus, CD8+ T cells initiate cytotoxic responses early in the infection by recognizing highly conserved epitopes of the viral nucleoprotein (NP) and matrix protein (M1). This leads to the direct lysis of virus-infected respiratory epithelial cells, significantly reducing viral load and the risk of severe disease (15, 16). These epitopes, due to their involvement in the assembly of the virus’s core structure, are less likely to undergo mutations, making CD8+ T cell responses have cross-strain protective potential. For example, cross-protection against different subtypes such as H1N1 and H5N1 can be mediated by these responses (17). An experiment using a single-cycle influenza vaccine (S-FLU) in female mice aged 6 to 8 weeks has confirmed that NP-specific CD8+ TRM cells deposited in the respiratory tract can recognize conserved epitopes from at least 12 different influenza strains. Their polyclonal nature ensures that mutations in a single epitope cannot completely escape immune surveillance (15, 18). At the same time, Th1 cells activate the antiviral state of alveolar macrophages by secreting cytokines such as IFN-γ. This not only directly inhibits the function of the viral polymerase but also upregulates the expression of MHC-I molecules on infected cells, enhancing the epitope presentation efficiency to CD8+ T cells (17, 19) (**Table 1**).

**Table 1:** (203–205).

Vaccine types	IFN-γ CD4+ (IU/mL) Day 14	IFN-γ CD4+ (IU/mL) Day 28	IFN-γ CD4+CD8+ (IU/mL) Day 14	IFN-γ CD4+CD8+ (IU/mL) Day 28	Cellular immunity strength (CD4^+^ T cells)	Cellular immunity strength (CD4^+^CD8^+^ T cells)	Immunity durability	Remarks
BBIBP (Inactivated Vaccine)	0.125 (0.053 - 0.453)	0.085 (0.030 - 0.238)	0.240 (0.060 - 0.615)	0.155 (0.053 - 0.410)	Weak	Weak	Short	Mainly induces humoral immunity
AZD1222 (Viral Vector Vaccine)	0.500 (0.188 - 1.173)	0.260 (0.090 - 0.710)	0.720 (0.325 - 1.690)	0.470 (0.170 - 1.430)	Moderate	Moderate	Moderate	Cellular immunity superior to inactivated vaccine but inferior to mRNA vaccine
BNT162b2 (mRNA vaccine)	1.250 (0.430 - 3.698)	0.790 (0.290 - 1.890)	2.020 (0.538 - 4.993)	1.260 (0.400 - 3.090)	Strongest	Strongest	Longest	Optimal balance between cellular and humoral immunity
CircRNA(Novel RNA Vaccine)	Strong (0.5–2.5 µg doses show better performance)	Unknown	Strong (0.5–2.5 µg better, no significant advantage at high dose)	Unknown	Moderate	Moderate	Unknown	Stronger immune response at low dose, no obvious advantage at higher dose
1mΨ-mRNA(Optimized mRNA Vaccine)	Strong (0.5–2.5 µg, better than CircRNA)	Unknown	Strong (0.5–2.5 µg, superior to CircRNA)	Unknown	High	High	Unknown	Immune response superior to CircRNA, close to BNT162b2
Aerosolized Ad5-nCoV (Mixed Dose Group)	41.57 (11.54 - 71.61)	–	81.42 (26.76 - 136.07)	–	High	High	Moderate	IL-5 and IL-13 significantly increased 14 days after 2nd dose
Aerosolized Ad5-nCoV (Single Dose Group)	45.31 (1.35 - 89.28)	–	13.04 (2.53 - 23.55)	–	Moderate	Moderate	Moderate	IFN-γ response higher than intramuscular group, significant Th1/Th2 immune responseas

#### 2.1.2 SARS-CoV-2

For SARS-CoV-2, the pressure to escape cellular immunity primarily comes from the CTL response targeting the S2 subunit of the spike protein (S protein), a region that plays a critical role in viral membrane fusion and, therefore, is constrained in terms of mutations (20, 21). Whole-genome T cell epitope scanning shows that approximately 60% of CD8+ T cell responses target non-structural proteins, such as ORF1ab. These regions are conserved in more than 85% of the Omicron variant (22, 23). Structural studies indicate that HLA-A*02:01-restricted epitopes of the spike protein (such as S269-277) can induce potent perforin-granzyme release. Even in the presence of key mutations like K417N and E484K, these epitopes maintain over 70% TCR recognition efficiency (24, 25). Respiratory TRM cells localize to the nasopharyngeal-associated lymphoid tissue (NALT) through CXCR3-mediated homing. Their phenotypic characteristics (CD69+CD103+) are similar to those of EBV-specific TRM cells, but their function is somewhat weaker. This may explain the phenomenon of repeated upper respiratory tract infections (26, 27). It is noteworthy that SARS-CoV-2 employs three molecular strategies to escape CTL surveillance: first, by using the NSP7 protein to mimic human T cell epitopes (for example, NSP795–102 shares 67% similarity with human PPIA), thereby achieving immune camouflage; second, by mutating the furin cleavage site of the spike protein to interfere with antigen processing; and third, by utilizing the ORF8-mediated MHC-I degradation pathway to systematically downregulate antigen presentation (25, 28, 29). Hybrid immunity (post-infection mRNA vaccination) induces the broadest TRM response. These cells exhibit unique transcriptional characteristics—high expression of TCF7 to maintain stem cell-like properties, and upregulation of KLF2 to enhance tissue residency. As a result, their survival time in bronchoalveolar lavage fluid is extended to more than 12 months after infection (30, 31). Single-cell TCR sequencing has also revealed that CD8+ T cell clones targeting SARS-CoV-2 exhibit a “layered cross-reactivity” feature: the foundational clones recognize conserved epitopes from seasonal coronaviruses, while the dominant clones specifically target unique epitopes of SARS-CoV-2. This dual recognition architecture significantly enhances the breadth of defense against variants (32, 33).

#### 2.1.3 RSV

The core challenge in the development of respiratory syncytial virus (RSV) vaccines lies in the immune pathology induced by a Th2-type immune bias. This bias is characterized by the excessive secretion of cytokines such as IL-4, IL-5, and IL-13 following vaccination, leading to pathological features such as eosinophil infiltration, airway hyperreactivity, and excessive mucus production (34, 35). This phenomenon was particularly evident in the 1960s formalin-inactivated RSV vaccine (FI-RSV) clinical trials, which manifested as exacerbated pulmonary inflammation and defective immune memory (36, 37). This pathological reaction may stem from the damage to viral surface proteins (e.g., PreF conformation) during vaccine preparation, leading to impaired innate immune recognition (e.g., TLR signaling) and aberrant Th2 polarization (38, 39). Notably, Th2 cytokines (e.g., IL-4, IL-5) not only promote eosinophil infiltration but also synergize with the IL-17 pathway—Th17 cells further amplify the inflammatory cascade through IL-17A/F secretion. This imbalance in T-cell subsets (Th2/Th17 dominance) has been confirmed in RSV infection models to exacerbate tissue damage (40–43). However, protective immunity requires coordinated T-cell responses: CD8+ T cells can directly clear virus-infected cells, while Th1-type responses (IFN-γ-dominated) and regulatory T cells (Tregs) can suppress excessive inflammation (44–46).

### 2.2 Durability and cross-protection of cellular immunity

#### 2.2.1 Memory T cell pool

In terms of cellular immunity durability and cross-protection, tissue-resident memory T cells (TRM) and central memory T cells (TCM) form a dual barrier against RSV reinfection. TRM cells (such as CD103+ CD69+ CD8+ T cells) reside in the respiratory mucosa, and their presence has been directly linked to reduced viral load in experimental RSV infections (47, 48). Their expansion depends on type I interferon signaling mediated by the MAVS and MyD88/TRIF pathways (49–51). In MAVS-deficient mice, TRM numbers can be restored to 80% of wild-type levels following IFN-α treatment (52). Single-cell RNA sequencing reveals that TCM cells initiate a transcriptional reprogramming process to differentiate into TRM cells within 2 days following intranasal LAIV booster immunization. This process involves the recruitment of chemokines dependent on CXCR3 and the upregulation of CD8+ TRM characteristic genes, such as Itgae and Cxcr6 (53). Circulating memory T cells maintain systemic immune surveillance through the TCM (CD62L+ CCR7+) and effector memory T cell (TEM) subsets. Under stimulation with RSV F protein peptides, PBMCs from uninfected individuals can generate a stable memory T cell response that lasts for more than 10 years (54). Long-lived TRM and circulating memory T cells (TCM) provide rapid recall responses upon re-exposure to the pathogen.

#### 2.2.2 Epitope conservancy

Epitope conservancy is key to achieving cross-protection. The preF conformation epitopes of the RSV F protein (such as antigenic sites Ø and V) are conserved by more than 95% between the A and B subtypes (55). In contrast, the mutation rate of T cell epitopes (such as M187–195 of the F protein) is only one-third of that of B cell epitopes (56). This makes it an ideal target for broad-spectrum vaccines. Multi-epitope vaccine design, by integrating HLA supertype epitopes of the F protein (such as the DRB104:01-restricted CD4+ epitope F254–268 and the HLA-A02:01-restricted CD8+ epitope F85-93), can simultaneously stimulate a balanced Th1/Th2 antibody response and CTL activity in mouse models (56, 57). It is worth noting that the multi-epitope display virus-like particles (VLP) based on the Round Leaf Bat Hepatitis Core Antigen (RBHcAg) can induce cross-neutralizing antibodies against RSV A2, B18537, and the clinical isolate hRSV/C-Tan/BJ 202301. The potency of these antibodies is comparable to that of approved vaccines (57, 58).

## 3 Vaccine-induced cellular immune responses in upper respiratory viruses

### 3.1 Cellular immune characteristics of different vaccine platforms

### 3.1.1 mRNA vaccine

mRNA vaccines carry the information encoding specific antigens (such as the spike protein of SARS-CoV-2), which are directly translated into antigens within cells, thereby activating a strong cellular immune response (59, 60). mRNA vaccines work by injecting mRNA that encodes the pathogen’s antigen, allowing the ribosomes within the cells to translate it into antigen proteins (61, 62). The synthesized antigens are processed by the cell’s endogenous pathways, generating peptides that bind to MHC class I molecules, which primarily activate CD8+ cytotoxic T cells (CTLs). This enables CD8+ T cells to recognize and bind to the MHC I-antigen complex, leading to activation, proliferation, and differentiation, and ultimately killing the infected cells (63–65). At the same time, some of the antigens are presented by antigen-presenting cells (such as dendritic cells) via MHC class II molecules, activating helper T cells (Th cells) (66). Furthermore, mRNA vaccines can induce a strong cytokine response, including pro-inflammatory cytokines (such as IL-12 and IFN-γ), which help enhance the activation and expansion of CD8+ T cells (67, 68). Particularly, under the regulation of cytokines secreted by CD4+ T cells, the generation of memory T cells is further promoted (65, 69). After the initial immune response, a portion of the CD8+ T cells will differentiate into memory T cells. The memory T cells induced by mRNA vaccines typically exhibit stronger functionality and longer survival. These memory T cells can respond rapidly when re-exposed to the same antigen, providing quick and effective immune protection (70). Therefore, mRNA vaccines can effectively induce CD8+ T cell and Th1 cell responses, providing strong cellular immune protection, making them particularly suitable for defending against intracellular pathogens and tumor cells (71). mRNA vaccines not only effectively induce humoral immunity (antibody production), but also activate potent cytotoxic T cells (CTL) via the MHC class I pathway, thereby combating viral infections. Since mRNA vaccines do not contain live viruses or viral proteins, there is no risk of infection. As a transient molecule, mRNA degrades quickly in the body and does not alter the host’s genes, ensuring a high level of safety (15).

#### 3.1.2 Inactivated vaccines

In contrast, traditional inactivated vaccines induce an immune response using inactivated virus particles. These vaccines inactivate the virus through physical or chemical methods, rendering it unable to cause infection (72). The vaccine contains inactivated whole viruses or viral components, and after injection, the viral antigens are directly presented, triggering an immune response. The antigens of inactivated vaccines enter cells primarily via the exogenous pathway, where they are taken up and processed, and then presented to CD4+ T cells through MHC class II molecules. This typically leads to the activation of Th2 cells. Th2 cells secrete cytokines that promote humoral immunity (such as IL-4 and IL-5), stimulating B cells to produce antibodies, particularly IgE antibodies, thereby enhancing the humoral immune response (73–75). However, the cellular immune response induced by inactivated vaccines is weaker because the antigens are mainly presented via MHC class II, which is insufficient to activate CD8+ T cells (76). Because inactivated vaccines typically induce a weaker cellular immune response, the quantity and functionality of the generated memory T cells may be insufficient. Compared to the strong immune responses induced by mRNA vaccines, inactivated vaccines may have lower durability and functionality of memory T cells (77). In addition, the persistence of antigens from inactivated vaccines in the body is relatively short, which may affect the formation and maintenance of memory T cells. The development and production cycles for traditional inactivated vaccines are longer, and the costs are higher. However, their safety profile is excellent, as the viruses in inactivated vaccines have lost their infectivity, ensuring that no infection is triggered after vaccination. Therefore, inactivated vaccines are highly safe and suitable for various populations, especially those with weakened immune systems (73, 78–80). The immune response induced by inactivated vaccines is primarily focused on humoral immunity, making them effective at inducing antibody responses, providing short-term protection against the virus. However, they are less effective at inducing cellular immunity, particularly in terms of activating CD8+ T cells (81).

#### 3.1.3 Viral vector vaccines

Adenoviral vaccines deliver target antigen genes into host cells using non-pathogenic viral vectors, efficiently activating multilayered cellular immune responses. The core mechanism lies in the ability of the viral vector to rapidly recognize receptors on the host cell surface, such as the coxsackievirus and adenovirus receptor (CAR) (82). The vector then enters the cell via endocytosis or membrane fusion, followed by antigen protein expression in the cytoplasm (83, 84). These antigens are degraded into peptides by the proteasome via the endogenous pathway, then bind to major histocompatibility complex class I (MHC-I) molecules and are presented on the cell surface, directly activating CD8^+^ T cells to differentiate into cytotoxic T lymphocytes (CTLs) (85). CTLs eliminate virus-infected cells directly by releasing perforin and granzymes, while also establishing a long-lasting pool of memory T cells to respond to future infections (86, 87). In addition, antigen-presenting cells (such as dendritic cells) transfer antigens to MHC class II molecules through the classical exogenous pathway, activating CD4^+^ T helper cells (Th1 subtype). These cells secrete cytokines such as interferon-gamma (IFN-γ), which further enhance the cytotoxic function of CD8^+^ T cells and promote macrophage activation (88, 89). The direct cytotoxicity of CD8^+^ CTLs and the immunoregulatory function of CD4^+^ Th1 cells work synergistically, not only enhancing antigen delivery efficiency (90, 91), but also inducing long-lasting immune memory by mimicking the natural infection pathway, thereby conferring (92).

#### 3.1.4 Novel nanoparticle vaccines

Novel nanoparticle vaccines efficiently activate cellular immune responses through their unique delivery systems and multifunctional designs. Nanoparticles (50–250 nm) enhance the uptake efficiency by antigen-presenting cells (APCs) through size-dependent effects and surface charge modulation, such as positive charge modifications (93, 94). Passive targeting relies on the enhanced permeability and retention (EPR) effect to accumulate in inflammatory or lymphoid tissues (94, 95). Active targeting involves surface modification with antibodies or peptide ligands (such as DC-SIGN ligands) to precisely recognize APC surface receptors (96). After the particles enter the cell, pH-sensitive materials [such as poly (β-amino esters)] disassemble in the acidic environment of the endosome, releasing the antigen into the cytoplasm and promoting cross-presentation by MHC-I molecules, which directly activates CD8^+^ T cells to differentiate into cytotoxic T lymphocytes (CTLs) (97, 98). If the antigen enters the lysosome, it activates CD4^+^ Th1 cells via MHC-II molecules, which secrete IFN-γ to enhance CTL function. Sustained-release designs (such as PLGA degradation control) can extend antigen exposure for up to 28 days, continuously stimulating the generation of memory T cells. Nanocarriers can co-deliver antigens and adjuvants (such as TLR3/7/9 agonists), enhancing synergistic effects through spatiotemporal synchronized delivery. Furthermore, nanoparticles surface-modified with mannose target APC surface C-type lectin receptors, improving drug delivery specificity, bioavailability, and therapeutic efficacy, while reducing off-target effects and systemic toxicity (99). Multifunctional nanocarriers achieve microenvironmental regulation through the co-delivery of immunomodulatory factors (100, 101). For example, PLGA nanoparticles loaded with OVA antigen and rapamycin (an mTOR inhibitor) can induce the differentiation of regulatory T cells (Tregs) and suppress Th17-mediated inflammatory responses. Th17-associated pro-inflammatory factors (IL-17, IL-1β, IL-12) are significantly reduced in the PLGA-Rapa treatment group, with some literature reporting a decrease in IL-17a of over 50% (102, 103). The ratio of anti-inflammatory factors (TGF-β1, IL-10) increases, with the secretion of TGF-β1 in the Rapa&P-50k group approximately doubling (104). In the OVA inflammation model, PLGA nanoparticles (such as IL10-AMNP) increase the proportion of Tregs and decrease the proportion of Th17 cells. The exact proportions vary depending on the model, but the trend remains consistent (105, 106). At the same time, it maintains antiviral CTL activity, achieving a balance between therapy and protection (107). This ‘immune switch’ design provides a precise intervention strategy for chronic infections or autoimmune diseases (**Table 2**).

**Table 2:** (206).

Vaccine type	Main antigens	Immune response	Advantages	Disadvantages
Inactivated Vaccine	Hemagglutinin (HA), Neuraminidase (NA)	Primarily induces antibody responses	Suitable for broad populations, high safety	Weak cellular immunity, protection easily affected by viral mutations
Live Attenuated Influenza Vaccine (LAIV)	Whole virus	Induces mucosal immunity, enhances T cell response	Mimics natural infection, enhances cross-protection	Not for immunocompromised individuals, efficacy varies by age
adjuvanted subunit vaccine (a/qdsA)	HA	Induces high-level antibodies and partial T cell response	Rapid strain updates for seasonal influenza	Durability of immunity needs assessment, requires cold chain
Virus-like Particle (VLP) Vaccine	HA, NA	Strong antibody and moderate T cell responses	Virus-like structure enhances immunogenicity	High production cost
Recombinant Protein Vaccine	HA, NA	Mainly induces antibody responses	No need for virus culture, avoids antigen drift	Possibly weaker immunogenicity
Nanoparticle Vaccine	HA, NP	Induces both antibody and T cell immunity	Enhances antigen stability and immune effectiveness	Still in research stage

### 3.2 Comparison of key indicators

#### 3.2.1 IFN-γ secretion levels

IFN-γ (interferon-gamma) is a key cytokine secreted by immune cells such as CD8^+^ T cells and NK cells, and its secretion level is an important indicator for evaluating vaccine-induced cellular immune responses. IFN-γ plays a central role in anti-infection and anti-tumor immunity by activating macrophages, enhancing antigen presentation, promoting Th1-type immune responses, and directly inhibiting viral replication or tumor growth (108–110). In vaccine immunization, high levels of IFN-γ secretion are typically associated with stronger immune protection, especially against pathogens that require cellular immunity for clearance, such as viruses or intracellular bacteria (111, 112). mThe IFN-γ secretion by CD8^+^ T cells induced by the RNA vaccine (BNT162b2) was significantly higher than that induced by the inactivated vaccine (BBIBP-CorV) (2.02 vs. 0.24 IU/mL), indicating that mRNA vaccines induce a stronger multifunctional CD8^+^ T cell response. mRNA vaccines are significantly superior to inactivated vaccines in activating Th1-type immune responses. Another study showed that CD8^+^ T cells induced by mRNA vaccines have a broader epitope coverage and are associated with reduced viral load in the upper respiratory tract (113, 114). This difference primarily arises from the distinct antigen designs and immune activation mechanisms of the two vaccines. mRNA vaccines (such as BNT162b2) encode a single target protein (such as the SARS-CoV-2 spike protein), efficiently activate dendritic cells, and directly present antigens through the MHC I pathway, thereby more strongly stimulating CD8^+^ T cell differentiation into effector cells and the secretion of IFN-γ (70, 112, 115). In contrast, although inactivated vaccines can induce CD8^+^ T cell responses targeting multiple viral proteins (such as S, N, and M proteins), their antigen presentation efficiency is lower and relies on cross-presentation pathways, leading to relatively weaker IFN-γ secretion by CD8^+^ T cells (70, 115, 116). Another comparative study showed that the IFN-γ secretion by CD8^+^ T cells induced by the mRNA vaccine is twice that of the inactivated vaccine (70). Moreover, the response is more focused on the S protein epitopes (115). In addition, the lipid nanoparticle (LNP) delivery system of mRNA vaccines may directly enhance T cell activation, further promoting IFN-γ production (117, 118). These differences suggest that mRNA vaccines have an advantage in stimulating Th1-type immunity and CD8^+^ T cell-mediated cytotoxicity.

#### 3.2.2 Memory T cell durability

Viral vector vaccines (such as AZD1222) maintain moderate CD8^+^ T cell activity 28 days after vaccination, while the response from inactivated vaccines rapidly declines. The advantage of viral vector vaccines (like AZD1222) in inducing memory T cell durability compared to inactivated vaccines is primarily due to their unique antigen presentation methods and immune activation mechanisms. Viral vector vaccines mimic the natural infection process, continuously expressing the target antigen [for example, adenoviral vectors can infect fibroblasts over the long term) (119)]. This results in the phenomenon of ‘memory inflation,’ where effector memory CD8^+^ T cells (TEM) continue to expand and maintain high numbers in peripheral tissues (120). This persistent antigen stimulation is achieved through two mechanisms: first, viral vectors (such as adenoviruses or cytomegalovirus vectors) can persist in host cells for extended periods and express the antigen at low levels (121, 122). Second, it preferentially targets antigen-presenting cells such as dendritic cells, promoting cross-presentation and activating CD8^+^ T cells (123). In contrast, the antigen of inactivated vaccines has a short-lived effect (78). It cannot form persistent stimulation, leading to a rapid decline in CD8^+^ T cell responses. Viral vector vaccines also enhance durability by inducing tissue-resident memory T cells (TRM), which remain distributed in various tissues months after vaccination (121, 124). In contrast, inactivated vaccines primarily induce circulating memory T cells (10). In addition, viral vector vaccines can more effectively activate cytokine pathways such as IL-15, promoting the early generation of memory precursor cells (such as Tscm) (125). These stem cell-like memory T cells have self-renewal capabilities and can maintain the memory pool for a long time (126). Phenotypically, viral vector-induced CD8^+^ T cells exhibit high expression of cytotoxic-related genes (127). They also exhibit higher functional affinity (128). The T cell subset distribution induced by inactivated vaccines is more limited (78). It is worth noting that adenovirus vector vaccines can also drive the expansion of innate-like CD8^+^ T cells by inducing host IL-15 production (122). This mechanism has not been reported in inactivated vaccines. Taken together, viral vector vaccines achieve more durable memory T cell maintenance compared to inactivated vaccines through multiple mechanisms, including persistent antigen exposure, tissue-resident memory formation, cytokine signaling activation, and the induction of stem cell-like memory subsets.

## 4 Comparison of local and systemic immunity in upper respiratory infections

The synergistic action of cellular and humoral immunity is crucial for respiratory virus vaccines. Current research indicates that although cellular immunity plays a key role in host protection (129), relying solely on a single immune mechanism often fails to confer comprehensive protection. For instance, the PIV5-vectored SARS-CoV-2 vaccine CVXGA1, administered intranasally, can simultaneously induce durable mucosal, cellular, and humoral immune responses, demonstrating the advantage of coordinated immunity (130). Studies have confirmed that when vaccines are able to simultaneously activate CD8^+^ T cell responses (cellular immunity) and neutralizing antibody production (humoral immunity), they achieve optimal protection against respiratory viruses, including influenza and SARS-CoV-2 variants (131–133). This synergistic relationship is particularly important in vaccine design, as respiratory viruses often evade pre-existing antibodies to escape immune defense (134, 135), whereas cellular immunity—such as CD8^+^ cytotoxic T cells—can recognize conserved viral epitopes and provide broad cross-protection (136, 137). Moreover, molecular docking studies suggest that ideal vaccine constructs should be capable of simultaneously engaging pattern recognition receptors like TLR3 and TLR8 to co-activate both arms of the immune response (138). Clinical observations have shown that while humoral immunity tends to wane over time following booster vaccination, cellular immune responses are more durable (139, 140), further highlighting the necessity of inducing both immune pathways. Therefore, the development of next-generation respiratory virus vaccines should focus on optimizing antigen design [e.g., co-delivery of spike and nucleocapsid proteins (141)], delivery platforms [e.g., adenoviral vectors (142)], and adjuvants [e.g., ARNAX (143)] to synergistically enhance mucosal IgA, systemic IgG, and T cell responses (144, 145), thereby providing more effective protection against immune-evading viral variants (146).

### 4.1 Synergy between local and systemic immunity

Local and systemic immunity do not exist in isolation; rather, they interact and coordinate through various mechanisms to form a more comprehensive and efficient immune response. In upper respiratory tract infections, the interaction between local TRM cells and systemic immune cells (such as CD8+ T cells, Th1 cells, etc.) plays a crucial role in enhancing the overall effectiveness of the immune response. The local immune response, particularly through the rapid response of TRM cells, can provide localized immune protection during the early stages of infection, while the systemic immune response strengthens the local immune function through the T cell response across the body (147). For example, local TRM cells can recruit and activate surrounding immune cells, such as neutrophils and macrophages, by secreting cytokines (e.g., interferon-γ and tumor necrosis factor-α), which help eliminate local infection sources. The local immune response can also act on immune organs such as lymph nodes to promote the activation and proliferation of systemic T cells, thereby further enhancing the breadth and persistence of the immune response (148). Experimental studies have shown that nasal immunization can promote the activation of the systemic immune system through local immune responses, and the systemic immune response can also enhance immune defense at the local infection site through memory T cells. Karaki et al., through mouse experiments, found that nasal spray vaccines could activate TRM cells in the respiratory tract, promoting local immune responses while enhancing the systemic immune response through cytokines, particularly the activation of CD8+ T cells and Th1 cells, ultimately improving the mouse’s defense against influenza virus (48).

### 4.2 The combination of mucosal vaccines and systemic vaccines

The immune characteristics of upper respiratory tract viruses dictate that an effective immunization strategy needs to balance both local and systemic immunity. Traditional intramuscular vaccines mainly activate systemic immunity and struggle to effectively induce mucosal immunity. The advantage of local mucosal vaccination is that it directly stimulates immune cells in the respiratory mucosa, rapidly inducing secretory IgA production. IgA can neutralize viruses, prevent them from attaching to and entering epithelial cells, and activate tissue-resident memory T cells (TRM), which rapidly respond after infection and help limit viral spread. Systemic intramuscular vaccines primarily generate a strong IgG antibody response and can induce circulating CD8^+^ cytotoxic T cells to clear infected cells. When these two vaccines are used together, they can establish a local barrier defense and mobilize systemic immune resources to provide multi-layered protection. Studies have shown that this strategy provides significant advantages in various viral models. For example, experiments demonstrate that combining adenovirus vector-based nasal vaccines and intramuscular vaccines significantly enhances protection against SARS-CoV-2 in mouse and non-human primate models. The nasal vaccine effectively induces IgA and TRM cell responses in the upper respiratory tract, while the intramuscular vaccine significantly boosts systemic neutralizing antibody levels and CD8^+^ T cell responses (149). Similarly, Animal experiments on mice studies on influenza virus have also shown that combining nasal immunization with inactivated virus and traditional intramuscular injection can provide more comprehensive immune coverage, reduce viral load, and prevent severe disease (150). In addition, this strategy is particularly important when addressing viral variants. Mucosal immune mechanisms demonstrate strong cross-protection against viral variants. For example, researchers genetically engineered two AD vectors for a trivalent vaccine (Tri: HuAd and Tri: ChAd) and extensively compared their immunogenicity and protective effects against both the ancestral and variant strains of SARS-CoV-2. The study found that trivalent ChAd vector vaccine delivered through the respiratory mucosa is the most effective next-generation COVID-19 vaccine strategy. Their research supports its further clinical development. On the other hand, systemic antibodies may be more sensitive to variant epitopes, and nearly all first-generation gene-based COVID-19 vaccines were designed for intramuscular delivery, expressing only the S protein (151). The combined strategy can better overcome the limitations of single vaccination methods. The integration of both local and systemic vaccination approaches is one of the key directions for future vaccine design.

### 4.3 The regulatory role of vaccine adjuvants on cellular immunity

The mechanisms by which adjuvants enhance T cell responses through activation of the innate immune system vary depending on their type. Aluminum salt adjuvants (Alum), one of the earliest adjuvants used, primarily function by activating the NLRP3 inflammasome to induce the secretion of IL-1β and IL-18, thereby promoting dendritic cell (DC) maturation and enhancing antigen presentation capacity. Additionally, Alum improves antigen persistence and delivery efficiency by forming antigen depots (152). Emulsifier-based adjuvants (e.g., MF59) enhance immune responses by promoting antigen presentation and activating innate immune cells, enabling antigen dose sparing, broader response range, and fewer immunizations. Their mechanisms include improving antigen distribution and uptake, inducing inflammatory chemokine production to attract monocytes and macrophages to the injection site, thereby promoting dendritic cell (DC) recruitment and activation (153). Pathogen-associated molecular pattern (PAMP)-based adjuvants, such as CpG oligonucleotides, activate innate immunity by binding to TLR9, inducing IL-12 secretion to promote Th1-type immune responses, and enhancing dendritic cell (DC) cross-presentation, enabling exogenous antigens to activate CD8+ T cells (154). Pathogen-associated molecular pattern (PAMP)-based adjuvants, such as CpG oligonucleotides, activate innate immunity by binding to TLR9, inducing IL-12 secretion to promote Th1-type immune responses, and enhancing dendritic cell (DC) cross-presentation, enabling exogenous antigens to activate CD8+ T cells (155).

Adjuvants primarily function by activating the innate immune system and modulating the activity of antigen-presenting cells (e.g., dendritic cells). Different types of adjuvants engage specific pattern recognition receptors (PRRs), such as Toll-like receptors (TLRs), retinoic acid-inducible gene I (RIG-I), melanoma differentiation-associated protein 5 (MDA5), and laboratory of genetics and physiology 2 (LGP2), to induce distinct cytokine environments, thereby shaping T-cell differentiation. For example, in Th1 responses, TLR3 or TLR9 ligands (e.g., Poly I:C or CpG oligonucleotides) promote IL-12 and IFN-γ production, enhancing cytotoxicity and antiviral immunity (156). For Th2 responses, aluminum salt-based adjuvants (e.g., alum) tend to stimulate macrophages containing large, persistent intracellular crystalline inclusions, a characteristic feature of muscle-infiltrating macrophages described in vaccine-injected animal models and recently reported in human macrophagic myofasciitis - myofasciitis (MMF) histological responses. Experiments by Gherardi, Verdier, et al. showed that macrophages were dominant in animals injected with aluminum hydroxide vaccines (mice, cynomolgus monkeys, rabbits, etc.), and the data obtained illustrated the critical role of this cell type in the physiological response to aluminum hydroxide-containing vaccines (157, 158). For Th17 responses, TGF-β, together with IL-6 and IL-21, promotes Th17 cell development. Adjuvants capable of activating IL-6, IL-23, and TGF-β, such as β-glucan molecules, can effectively induce Th17 cell differentiation, suitable for immune responses against fungal or extracellular bacterial infections (159). Optimizing specific immune response directions requires comprehensive consideration of pathogen characteristics and the desired effector mechanisms, achieving enhanced specific immune pathways through selective adjuvant combinations.

### 4.4 Comprehensive enhancement of cellular immunity

Enhancing the formation and function of tissue-resident memory T cells (TRM) while increasing the quantity and activity of systemic memory T cells to achieve comprehensive immune protection is a key topic in current immunology research. In local immunity, promoting TRM formation and function requires targeted optimization of the local microenvironment and signaling molecules. TRM generation relies on specific tissue-retention signals (160), such as cytokines like TGF-β and IL-15, which drive the formation and function of resident cells in local tissues by regulating the expression of TRM signature molecules (e.g., CD69 and CD103). TGF-β can enhance TRM adhesion to epithelial cells by promoting CD103 expression, thereby strengthening their retention capacity, while IL-15 improves TRM survival by supporting metabolic adaptability and anti-apoptotic signaling (161, 162). Studies show that in specific environments like adipose tissue, TRM adapt to local nutritional conditions by upregulating genes related to fatty acid oxidation (e.g., Cpt1a and Pparg), further enhancing their persistence and effector functions. Local immune enhancement can be optimized through vaccine delivery strategies (163, 164), such as local injection of TGF-β or IL-15 agonists, to accelerate TRM generation and improve their immune response to local pathogens. Systemic immune enhancement focuses on increasing the quantity and activity of circulating memory T cells (TCM) and effector memory T cells (TEM) to provide broad systemic protection (165) (162). A key strategy for systemic immune enhancement is inducing a high-quality memory T cell pool through vaccination. Using genetically modified antigen vaccines or adjuvants (e.g., TLR agonists or IL-12) can significantly enhance T cell proliferation and effector functions. IL-12 activates T cell effector functions via the STAT4 signaling pathway and promotes their differentiation into TEM. Additionally, systemic immunity can be improved by facilitating the interconversion between circulating and resident T cells. TEM cells can enter local tissues and, upon receiving TGF-β and IL-15 signals, convert into TRM, thereby establishing long-term immune memory locally (166, 167). Local immunity promotes TRM generation and persistence by optimizing the microenvironment, while systemic immunity ensures broad defense by expanding the memory T cell pool. A combined strategy, utilizing local and systemic cytokine co-delivery or optimized vaccine platforms, can significantly enhance the functions of TRM and TCM/TEM. This ultimately achieves more comprehensive and durable protection against pathogens.

## 5 Clinical challenges

### 5.1 The impact of immune escape and variant strains on vaccine efficacy

The emergence of viral variants can lead to changes in surface antigenicity through antigenic drift or antigenic shift, thereby impacting vaccine efficacy. In terms of cellular immunity, alterations in T cell epitopes are a key factor (168). SARS-CoV-2 variants carry multiple mutations, particularly concentrated in the receptor-binding domain (RBD) and antigenic epitope regions of the spike (S) protein, which may lead to a reduced recognition efficiency of certain mutated epitopes by vaccine-induced T cells (169–171). For example, the extensive mutations in the Omicron variant not only weaken the neutralizing antibody efficacy but also affect certain CD8+ T cell epitopes, thus reducing the cytotoxic T cell killing ability. Additionally, CD4+ T cell responsiveness to variants may be diminished if the mutated epitopes cannot be effectively presented by antigen-presenting cells. Despite the strong robustness of cellular immunity, the ability to recognize conserved viral epitopes (such as the S2 region of the S protein or the nucleocapsid (N) and membrane (M) proteins) still exists across different variants, but mutations in these conserved epitopes could further threaten the stability of cellular immunity (172). For rapidly mutating viruses, such as the influenza virus and respiratory syncytial virus (RSV), similar mutations may also alter key epitopes, leading to a reduction in vaccine efficacy.

### 5.2 Differences in vaccine efficacy among immunocompromised populations

#### 5.2.1 Age and immune response

There are significant differences in cellular immune responses to vaccination across different age groups. Adults generally exhibit the strongest T-cell responses, while infants and the elderly show lower reactivity due to the unique states of their immune systems. In infants, the immune system is still under development, with a higher proportion of naive T cells that are functionally immature, and limited capacity of antigen-presenting cells. This results in a significant reduction in the proliferation and effector function of CD4+ and CD8+ T cells after vaccination compared to adults. In contrast, elderly individuals experience immunosenescence, characterized by a significant reduction in the naive T cell pool. Immunosenescence weakens T cell activation and sensitivity to antigens, reduces the diversity of memory T cells, and chronic inflammation (inflammaging) further suppresses vaccine-induced cellular immune responses, thereby significantly affecting vaccine efficacy in older adults (173–175). Additionally, studies have found that elderly individuals exhibit significantly lower CD8+ T cell reactivity after receiving the pertussis vaccine compared to younger individuals, and the functionality of effector T cells is also impaired (175, 176). A study on age and immune response (with CMV infection as an immune-related phenotype) showed that the CMV positivity rate was 42.86% in young individuals with an average age of 25.3 years (aged 20–31 years at enrollment) and 55.56% in elderly individuals with an average age of 76.96 years (aged 60–96 years at enrollment). According to the IMM-AGE score, the immune aging index of a 90-year-old was approximately 3.2 times higher than that of a 66–67-year-old. The final results indicated that immune senescence begins in mid-adulthood (ages 40–60) (177). A study by Wu et al. showed that the antibody titers (GMT values) induced by the mRNA vaccine were significantly higher in younger individuals compared to the elderly. Specifically, 28 days after vaccination, the GMT for younger individuals was 302.9, while the GMT for the elderly was 173.2 (178). These studies suggest that the immune system status at different age stages significantly affects the cellular immune response induced by vaccines. Both infants and the elderly face greater immune response deficiencies, highlighting the need to specifically optimize vaccination strategies for these populations.

#### 5.2.2 Challenges in immunosuppressed and chronic disease patients

Immunocompromised and chronic disease patients often exhibit weakened cellular immune responses following vaccination, which poses significant challenges to vaccine efficacy and clinical application. In immunocompromised patients, such as those who have undergone organ transplants, the long-term use of immunosuppressive drugs significantly reduces their T-cell-mediated immune response, leading to a weaker vaccine-induced cytotoxic T lymphocyte (CTL) response (179, 180). Studies have shown that kidney transplant recipients exhibit CD4+ and CD8+ T cell responses after receiving the mRNA COVID-19 vaccine that are only 30%-50% of those seen in healthy individuals. Chronic kidney disease patients also face similar issues, with research indicating a reduction in memory T cell function and lower protection after vaccination compared to healthy controls (181). In chronic disease patients, such as those with diabetes, vaccine-induced cellular immune responses are also significantly affected due to chronic inflammation and immune system dysfunction (182). These characteristics lead to a higher infection risk for these patient groups when facing emerging viruses like SARS-CoV-2. Even after completing vaccination, additional preventive strategies, such as booster doses and passive immunotherapy, should be considered. Yang indicated that the third or fourth dose of the SARS-CoV-2 vaccine significantly improved the immunogenicity rate in dialysis patients, and this beneficial effect was not altered by vaccine type (same or different immunogenic vaccine), dialysis method (HD or PD), or prior low response after two doses of the vaccine (183).

## 6 Future research directions

### 6.1 Emerging immuno-monitoring technologies

Emerging immuno-monitoring technologies, such as single-cell RNA sequencing (scRNA-seq) and multi-color flow cytometry, play a crucial role in studying vaccine-induced cellular immunity. Single-cell RNA sequencing (scRNA-seq) enables gene expression analysis at the single-cell level, revealing transcriptomic changes in immune cells before and after vaccination, and helping identify specific cell subpopulations and their functional states. Weng et a. us’d scRNA-seq technology to analyze peripheral blood mononuclear cells (PBMCs) from convalescent COVID-19 patients, aiming to uncover dynamic changes in immune responses and the characteristics of cell subpopulations. The study collected PBMC samples from 10 COVID-19 patients and healthy controls, isolated the cells through Ficoll-Hypaque density gradient centrifugation, and constructed single-cell RNA libraries using the 10x Genomics Chromium Single Cell 5′ system. After high-throughput sequencing, the data were processed using Cell Ranger software, barcode labeling was applied, and data integration, normalization, and dimensionality reduction were performed using the Seurat software package. The study revealed the remodeling process of the immune system in convalescent patients, including an increase in the proportion of CD4+ and CD8+ memory T cells, a tendency of B cells to differentiate into plasma cells, and specific changes in the function of monocytes and NK cells. This provided valuable insights into immune recovery following COVID-19 infection (184–187). Multi-color flow cytometry, by using a variety of fluorescent markers, can identify and characterize cell subpopulations of interest. It can rapidly analyze tens of thousands of cells per second and, through cell sorting, isolate pure, viable cell populations, enabling precise characterization and quantitative analysis of different immune cell groups (188, 189). Guo et al. used a 21-color flow cytometry panel to analyze immune cell subpopulations in human non-small cell lung cancer (NSCLC) tissues. They assessed the proportions of different cell subpopulations in the lung cancer tissues, as well as the immune phenotypes and differentiation states of major cell populations. The successfully established 21-color flow cytometry protocol is applicable for detecting PBMCs and NSCLC tissue samples, providing an effective new approach for monitoring the immune microenvironment in lung cancer (190). The combination of these technologies provides powerful tools for a deeper understanding of the mechanisms behind vaccine-induced immune responses.

### 6.2 Personalized vaccination strategy

Personalized vaccination strategies are an important development direction in modern medicine, aiming to design more precise and efficient vaccination plans by analyzing an individual’s immune background. An individual’s immune response to vaccines can vary significantly due to differences in genetic background, age, gender, health status, and environmental factors. In practical applications, personalized vaccination strategies not only enhance an individual’s immunity but also effectively improve overall vaccination rates. Particularly for high-risk groups, understanding and utilizing the characteristics of breakthrough infections can help public health officials create more targeted vaccination plans to better protect these vulnerable populations (191, 192). As the COVID-19 pandemic continues, the implementation of personalized vaccination strategies will be key to improving vaccine effectiveness. Thus, personalized vaccination strategies have gradually become a focus of vaccine research. The core idea is that the genomic instability of tumors leads to the production of tumor-specific neoantigens, which are absent in normal tissues and serve as ideal targets for personalized vaccines (193–195). High-throughput sequencing and bioinformatics analysis can be used to identify specific neoantigens in a patient’s tumor, providing the basis for personalized vaccine design. Vaccines can be tailored based on the patient’s tumor mutation profile, using mutation hotspots and predictive algorithms to select candidate neoantigens that effectively activate CD8+ and CD4+ T cells (196–199). Additionally, mRNA vaccines and peptide vaccines are the main technological platforms for personalized vaccines, as they are highly flexible and can quickly incorporate patient-specific neoantigens for production. To further enhance efficacy, personalized vaccines can be combined with immune checkpoint inhibitors or other immunotherapies, using synergistic effects to amplify anti-tumor immunity (200). In recent years, some literature has supported the feasibility and potential of personalized vaccination strategies. It has demonstrated the safety of two types of personalized cancer vaccines in small-scale human trials, which showed positive clinical responses in high-risk melanoma patients. These results confirm that vaccines specifically designed based on a patient’s individual cancer mutations are feasible and safe in clinical practice. They also provide valuable insights for the development of personalized cancer immunotherapy strategies (201, 202).

## 7 Conclusion

Different vaccines for upper respiratory viruses exhibit significant differences in inducing cellular immune responses. Live attenuated vaccines typically simulate natural infection and induce a stronger T cell immune response, particularly in the activation of effector CD8+ T cells. In contrast, inactivated vaccines and subunit vaccines mainly enhance the activity and auxiliary functions of antigen-specific CD4+ T cells to support the immune response. Emerging nucleic acid vaccines (mRNA and DNA) can also significantly induce strong and durable cellular immune responses through efficient antigen expression. The ability of different vaccines to induce mucosal immunity (such as tissue-resident memory T cells, TRM) also varies, which directly impacts the local defense against viruses. Future vaccine design and research should focus on the following key directions: optimizing mucosal immune responses, studying how to enhance vaccine-induced TRM cells and other mucosal immune effects to better prevent virus replication and transmission in the upper respiratory tract; developing multi-target, broad-spectrum antigens targeting high mutation regions of viruses to improve immune protection against variant strains; exploring the specific mechanisms by which different vaccine platforms induce cellular immunity, especially the generation and maintenance of T cell memory; designing personalized vaccines based on host immunology and genetic data to meet the immune needs of different populations; exploring the combined use of different types of vaccines (such as live attenuated vaccines and nucleic acid vaccines) or supplementation with immunomodulators to enhance the breadth and strength of immune effects. These directions are expected to drive the development of next-generation upper respiratory virus vaccines and provide scientific evidence and technical support for addressing viral mutations and potential future pandemics.
